# SOCS3 inhibiting migration of A549 cells correlates with PYK2 signaling in vitro

**DOI:** 10.1186/1471-2407-8-150

**Published:** 2008-05-28

**Authors:** Siyang Zhang, Dawei Guo, Lili Jiang, Qingfu Zhang, Xueshan Qiu, Enhua Wang

**Affiliations:** 1 Department of Pathology, College of Basic Medical Sciences, China Medical University, Shenyang, PR China; 2 Department of Pathology, the First Affiliated Hospital of China Medical University, Shenyang, PR China; 3 Department of Surgery, the Fourth Affiliated Hospital of China Medical University, Shenyang, PR China

## Abstract

**Background:**

Suppressor of cytokine signaling 3 (SOCS3) is considered to inhibit cytokine responses and play a negative role in migration of various cells. Proline-rich tyrosine kinase 2 (PYK2) is a non-receptor kinase and has been found crucial to cell motility. However, little is known about whether SOCS3 could regulate PYK2 pro-migratory function in lung cancer.

**Methods:**

The methylation status of SOCS3 was investigated in HBE and A549 cell lines by methylation-specific PCR. A549 cells were either treated with a demethylation agent 5-aza-2'-deoxycytidine or transfected with three SOCS3 mutants with various functional domains deleted. Besides, cells were pretreated with a proteasome inhibitor β-lactacystin where indicated. The effects of SOCS3 up-regulation on PYK2 expression, PYK2 and ERK1/2 phosphorylations were assessed by western blot using indicated antibodies. RT-PCR was used to estimate PYK2 mRNA levels. Transwell experiments were performed to evaluate cell migration.

**Results:**

SOCS3 expression was found impaired in A549 cells and higher PYK2 activity was correlated with enhanced cell migration. We identified that SOCS3 was aberrantly methylated in the exon 2, and 5-aza-2'-deoxycytidine restored SOCS3 expression. Reactivation of SOCS3 attenuated PYK2 expression and phosphorylation, cell migration was inhibited as well. Transfection studies indicated that exogenous SOCS3 interacted with PYK2, and both the Src homology 2 (SH2) and the kinase inhibitory region (KIR) domains of SOCS3 contributed to PYK2 binding. Furthermore, SOCS3 was found to inhibit PYK2-associated ERK1/2 activity in A549 cells. SOCS3 possibly promoted degradation of PYK2 in a SOCS-box-dependent manner and interfered with PYK2-related signaling events, such as cell migration.

**Conclusion:**

These data indicate that SOCS3 negatively regulates cell motility and decreased SOCS3 induced by methylation may confer a migration advantage to A549 cells. These results also suggest a negative role of SOCS3 in PYK2 signaling, and a previously unidentified regulatory mechanism for PYK2 function.

## Background

Proline-rich tyrosine kinase 2 (PYK2) is a ubiquitously expressed non-receptor protein tyrosine kinase that contributes to integrate signals from receptor tyrosine kinases and intracellular signaling molecules in processes such as cell survival [1], proliferation [2] and motility [3]. PYK2 is hard to detect in early embryos [4], however macrophages from PYK2-knockout embryos demonstrate severe migration and function defects [5], and loss of Pyk2 kinase activity induces prostate cells to acquire a malignant, migratory phenotype [6]. Conversely, enhanced PYK2 signaling facilitates cell survival in an anchorage-independent manner [7], and promotes cell motility [8]. Moreover, inhibition of Pyk2 mostly results in decreased cell migration [9,10]. Along these lines, report has been shown that PYK2 expression and/or its activity are up-regulated in invasive cancer cells [11]. Ligand binding of receptor tyrosine kinase results in autophosphorylation at Tyr402, which serves as a binding site for several Src homology 2 (SH2) domain-containing proteins, such as Src kinase. The PYK2-Src complex leads to further phosphorylation of PYK2, resulting in activation of a number of cytoskeleton-linked proteins, which transduce signals to downstream pathways, such as the mitogen-activated protein (MAP) kinase cascades, and leads to control of cell survival [12], proliferation [13], and migration [14]. At present, little is known about the molecular mechanisms that negatively regulate PYK2 function in lung cancer.

Suppressor of cytokine signaling 3 (SOCS3) is a member of SOCS family and acts in a feedback loop to inhibit cytokine responses and STAT3 activation in various cells [15-17]. Evidence has indicated that SOCS3 regulates other signaling pathways as well [18,19]. SOCS3 expression is suppressed due to aberrant methylation in its promoter region, which frequently occurs in a variety of human tumors [20,21]. The SOCS3 protein is structurally composed of distinct functional domains, including a N-terminal kinase inhibitory region, a central SH2 domain and a C-terminal homologous region termed SOCS-box. In many cases, SOCS3 interacts with phosphorylated tyrosine residues mediated by the SH2 and KIR domains, and negatively regulates activities of tyrosine kinases [22,23]. The SOCS-box may act as a bridge to mediate degradation of target proteins that interact with SOCS3-SH2 by the proteasome pathway. Researches have proved that SOCS3 not only regulates the STAT3 signaling pathway [24] but also the FAK tyrosine kinase [25].

We report here that SOCS3 was down-regulated due to methylation in the exon 2 and reactivated after 5-aza-2'-deoxycytidine treatment in A549 cells. SOCS3 was found to interact with PYK2 by its SH2 and KIR domains, and facilitate PYK2 degradation via proteasome pathway, and to inhibit Tyr402 and ERK1/2 phosphorylations as well as PYK2-associated migration of A549 cells. These results identify a potential role of SOCS3 in regulating PYK2 signaling.

## Methods

### Cells treatment and transfection

**Figure 1:**
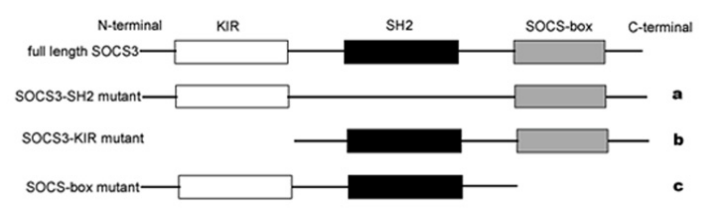
**Schematic representations of SOCS3 and three mutants with distinct functional domains deleted.** The SOCS3 protein is structurally characterized by three distinct functional domains that include a N-terminal kinase inhibitory region (white), a central SH2 domain (black) and a C-terminal homologous region SOCS-box (gray). (a) The SOCS3-SH2 mutant is a full length SOCS3 without the SH2 region, consisting of the KIR and SOCS-box domains. (b) The KIR region is knocked out in the SOCS3-KIR mutant, with the SH2 and SOCS-box domains remained. (c) The SOCS-box mutant contains the SH2 and KIR domains, with the SOCS-box deleted. A myc tag was added to each of these SOCS3 mutants, and exogenous SOCS3 mutants were represented by the detection of myc tag.

HBE cells (human bronchial epithelial) were grown in RPMI 1640, and A549 cells (human lung adenocarcinoma) were cultured in DMEM, both supplemented with 10% fetal bovine serum. The SOCS3-SH2, SOCS3-KIR and SOCS-box mutants were kind gifts from Dr. Kristiina Vuori (Burnham Institute, CA, USA). The SOCS3-SH2 mutant is a full length SOCS3 without the SH2 region, consisting of the KIR and SOCS-box domains. The KIR domain is knocked out in the SOCS3-KIR mutant, with the SH2 and SOCS-box regions remained. And the SOCS-box mutant contains the SH2 and KIR domains, with the SOCS-box deleted. A myc tag was added to each of these SOCS3 mutants, and exogenous SOCS3 mutants were represented by the detection of myc tag. The schematic representations of the three SOCS3 mutants with distinct functional domains deleted were shown in Fig. 1. For the reactivation study, A549 cells (50–60% confluence) were treated with 10 μM 5-aza-2'-deoxycytidine (Sigma) for 6 days, and medium was changed every other day. DNA and RNA as well as protein were extracted and analyzed as described below. To exogenously express SOCS3 for subsequent analysis, A549 cells (80–90% confluence) were transfected with either SOCS3 mutants or pcDNA3 vector for 48 h using Lipofectamin2000 (Invitrogen), according to the manufacturer's instructions. Where indicated, the proteasome inhibitor, β-lactacystin (Santa Cruz) at 25 μM was added to cells and maintained throughout the experiments.

### Methylation-specific PCR

Methylation-specific PCR (MSP) was carried out as described previously [26]. The methylation-specific and unmethylation-specific primers were designed using the Methyl Primer Express software v1.0 (ABI). Sequences of methylation-specific primers in the exon 2 of SOCS3 were 5'-TTC GAG GTG TTC GAG TAG TC-3' (forward) and 5'-AAC GAT CTT CCG ACA AAA AT-3' (reverse); sequences of unmethylation-specific primers were 5'-TTT TTT GAG GTG TTT GAG TAG TT-3' (forward) and 5'-AAC AAT CTT CCA ACA AAA ATA CT-3' (reverse), and the lengths of PCR products were both 159 bp. PCR was carried out by an initial denaturation step at 95°C for 10 min, followed by 40 cycles of denaturation at 95°C for 40 s, annealing at 63°C (methylation) or 62°C (unmethylation) for 40 s, and elongation at 72°C for 40 s. Cycling was completed by a final elongation step at 72°C for 10 min. Sequences of the other two MSP primer sets for exon 1 and intron 1 of SOCS3 are available upon request.

### RT-PCR analysis

Total RNA was prepared using Trizol (Invitrogen). cDNA was generated by reverse transcription of 1 μg total RNA using Reverse Transcriptase M-MLV (Takara). The primer sequences of PYK2 were 5'-AGA TTC CCG ACG AAA CCC-3' (forward) and 5'-CAC GGC GAA CAT CCA GAC-3' (reverse), and that of β-actin were 5'-AAA TCG TGC GTG ACA TTA A-3' (forward) and 5'-CTCGTCATACTCCTGCTTG-3' (reverse). The lengths of PYK2 and β-actin PCR products were 629 bp and 455 bp respectively. PCR was carried out by an initial denaturation step at 94°C for 5 min, followed by 35 cycles of denaturation at 94°C for 40 s, annealing at 54°C for 40 s, and elongation at 72°C for 40 s. Cycling was completed by a final elongation step at 72°C for 5 min. The PCR for β-actin was performed in the same manner as PYK2, except that 30 cycles of annealing at 50°C was used. The EC3 Imaging system (UVP Inc.) was used to catch up the specific bands, and the optical density of each band was measured using the Image J software.

### Western blot and co-immunoprecipitation analysis

Cells were washed twice with ice-cold phosphate buffered saline (PBS) and lysed in M-PER reagent (PIERCE) containing 1 mM PMSF and phosphatase inhibitors. The following primary antibodies were used in this study: mouse monoclonal anti-myc tag (Applygen, China), rabbit polyclonal anti-SOCS3, anti-PYK2, anti-p-Tyr40, anti-β-actin, and mouse monoclonal anti-p-ERK1/2 antibodies (all from Santa Cruz). Cells were lysed at 6 d after 5-aza-2'-deoxycytidine treatment or 48 h post-transfection, and the lysates were subjected to SDS-PAGE followed by western blot analysis. Where indicated, cells were pretreated with β-lactacystin 4 h before 5-aza-2'-deoxycytidine treatment or transfection and maintained throughout the experiments. The co-immunoprecipitation study was performed using the profound mammalian c-myc tag IP/Co-IP kit (PIERCE), according to the manufacturer's protocols. Clarified cell extracts were preincubated with agarose coupled with anti-myc tag antibody at 4°C overnight followed by SDS-PAGE and detected with anti-PYK2 antibody. The EC3 Imaging system (UVP Inc.) was used to catch up the specific bands, and the optical density of each band was measured using the Image J software.

### Transwell cell migration assay

Cell migration assay was performed using a 24-well Transwell chamber (Costar). At 6 d following 5-aza-2'-deoxycytidine treatment, cells (1 × 10^4^) were detached and seeded in the upper chamber of a 8 μm pore size insert in the 24-well plate and cultured for another 12 h. At 48 h after transfection, cells were treated similarly as described above. Cells were allowed to migrate forward to DMEM containing 15% FBS in the bottom chamber. The non-migratory cells on the upper membrane surface were removed with a cotton tip, and the migratory cells attached to the lower membrane surface were fixed with 4% paraformaldehyde and stained with hematoxylin. The number of migrated cells was counted in five randomly selected high power fields under microscope. Data presented are representative of three individual wells.

### Statistical analysis

The SPSS 13.0 software was applied to complete data processing. Independent-samples t-test was used to evaluate the differences of optical density (OD) values or numbers of migrated cells between groups with various treatments. All data were represented as mean ± SD of three independent experiments. Results were considered statistically significant when the p-value was less than 0.05 [see Additional file 1].

## Results

### Up-regulation of PYK2 expression, Tyr402 and ERK1/2 phosphorylations, as well as cell migration in A549 cells

**Figure 2:**
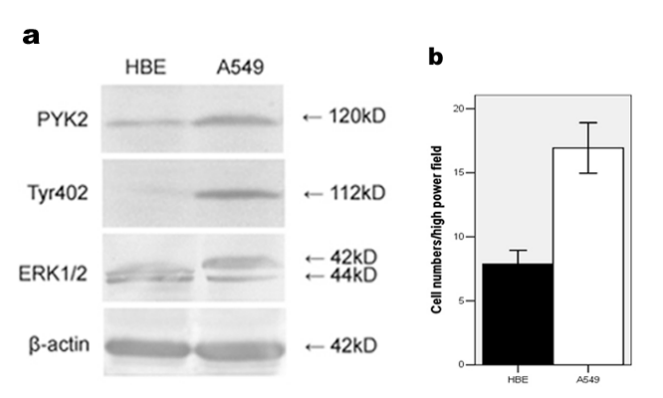
**PYK2 expression, Tyr402 and ERK1/2 phosphorylations, as well as cell migration in HBE and A549 cells.** (a) PYK2 expression, Tyr402 and ERK1/2 phosphorylations in HBE and A549 cells by western blot. (b) Comparisons of migratory abilities between HBE and A549 cells by Transwell assay. The data are representative of three individual experiments.

PYK2 expression and activation, as indicated by Tyr402 phosphorylation, and ERK1/2 phosphorylation were evaluated in HBE and A549 cells respectively. As normal lung cells, HBE cells expressed a low level of PYK2 with an undetectable phosphorylated Tyr402. A basal ERK1/2 phosphorylation was also observed in HBE cells. However, we found much higher expression of PYK2 as well as Tyr402 and ERK1/2 phosphorylations in A549 cells (p < 0.05, Fig. 2a). Additionally, more migrated A549 cells (16.9 ± 3.6) at 12 h were detected than HBE cells (7.9 ± 1.9, p < 0.05, Fig. 2b) by Transwell assay.

### Methylation-associated down-regulation of SOCS3 in A549 cells

**Figure 3:**
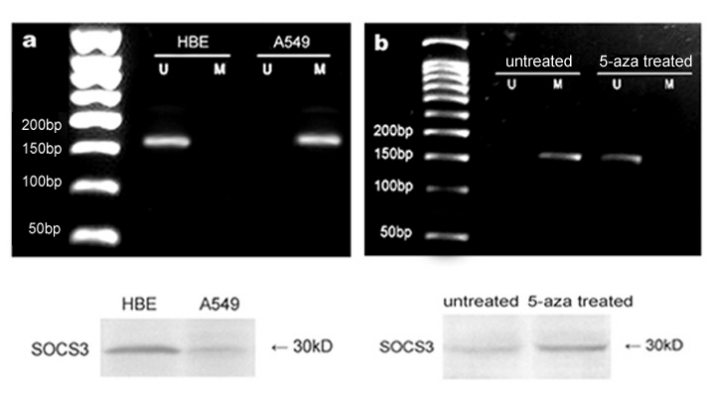
**Methylation status and expression of SOCS3 in HBE and A549 cells.** (a) Methylation status of SOCS3 in HBE and A549 cells, using the primer set designed in the exon 2, and detection of SOCS3 expression by western blot. The observed bands in lane M are methylated 159 bp products with methylation-specific primers and that in lane U are unmethylated 159 bp products with unmethylation-specific primers. Methylation was found in A549 cells but not in HBE cells and HBE cells expressed higher level of SOCS3. (b) The visible band in lane U appeared in A549 cells and SOCS3 was reactivated by the treatment of 5-aza-2'-deoxycytidine. The results are representative of three independent experiments.

The expression of SOCS3 was examined in HBE and A549 cells. We found that SOCS3 expression was much lower in A549 than HBE cells (p < 0.05, Fig. 3a). Researches on human cancers have indicated that down-regulation of SOCS3 was correlated with aberrant methylation. Thus, in our studies, three methylation- and non-methylation-specific primer sets were used to detect methylation status of SOCS3 in the exon 1, intron 1 and exon 2 in A549 cells respectively. As shown in Fig. 3a, methylation of SOCS3 in the exon 2, which lies in a region of 92–250 bp downstream to the translation start site, was detected in A549 cells, although no detectable methylation was found in the noncoding exon 1 and intron 1 (data not shown). Moreover, no methylation was observed in HBE cells as well. Therefore, methylation in the exon 2 was correlated with the decrease of SOCS3 expression in A549 cells. The non-methylation specific amplification instead of methylation was detected after the demethylation agent 5-aza-2'-deoxycytidine treatment, and SOCS3 expression was restored (p < 0.05, Fig. 3b), which further demonstrated that SOCS3 was methylated in A549 cells.

### Up-regulation of SOCS3 inhibited cell migration and associated PYK2 expression, Tyr402 and ERK1/2 phosphorylations in A549 cells

**Figure 4:**
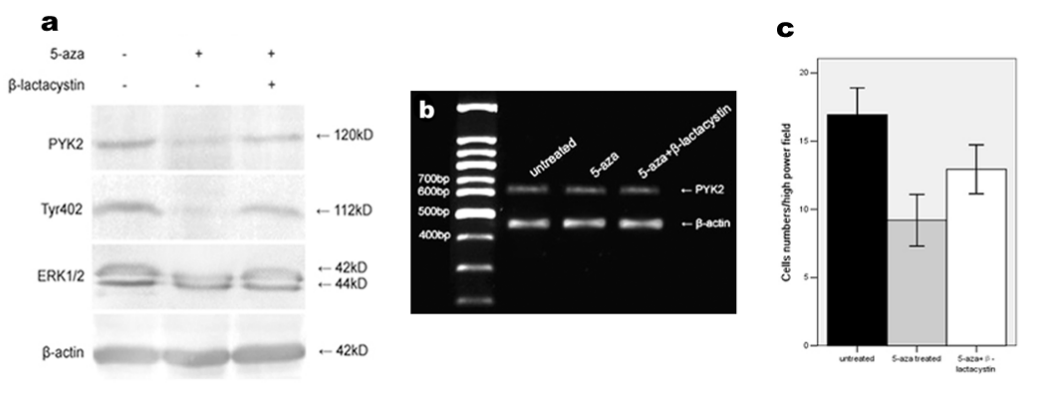
**Up-regulation of SOCS3 by 5-aza-2'-deoxycytidine treatment inhibited cell migration and associated PYK2 expression, Tyr402 and ERK1/2 phosphorylations.** (a) Decreased PYK2 expression, Tyr402 and ERK1/2 activations and migration of A549 cells were observed by SOCS3 up-regulation. A549 cells were treated with 5-aza-2'-deoxycytidine for 6 d, and cell lysates were analysed by western blot using the indicated antibodies. Elevated PYK2 expression, Tyr402 and ERK1/2 phosphorylations were found by the pretreatment of β-lactacystin. (b) PYK2 mRNA levels remained invariable regardless of treatment or not. The amplified PYK2 products were 629 bp in length. β-actin amplification demonstrated the consistency of PT-PCR. (c) Cell migration was suppressed by SOCS3 restoration, and the β-lactacystin pretreatment facilitated cell migration to some extent. The values are means of three replicates.

To investigate whether SOCS3 involves in regulating cell migration, the number of migrated A549 cells with 5-aza-2'-deoxycytidine treatment or not was evaluated. The numbers of migrated cells with or without 5-aza-2'-deoxycytidine treated and with β-lactacystin pretreatment were 9.2 ± 3.4, 16.9 ± 3.6 and 13.4 ± 3.3, respectively (p < 0.05, Fig. 4c). We also identified that PYK2 expression, Tyr402 and ERK1/2 phosphorylations were suppressed upon SOCS3 up-regulation by 5-aza-2'-deoxycytidine treatment (p < 0.05, Fig. 4a) and the decreased Tyr402 phosphorylation was in parallel with the reduced PYK2 expression which was possibly attributed to the process of proteolytic degradation, because elevated PYK2 expression was detected in cells pretreated with the proteasome inhibitor β-lactacystin (p < 0.05, Fig. 4a), and PYK2 mRNA levels were unaffected (p > 0.05, Fig. 4b).

### Roles of SOCS3 mutants in regulating PYK2 expression, Tyr402 and ERK1/2 activations and migration of A549 cells

Concurred with PYK2 expression, cells with the SH2 domain deleted SOCS3 showed phosphorylations of Tyr402 and ERK1/2 were identical to that in the vector alone-transfected cells (p > 0.05, data not shown). The numbers of migrated cells transfected with vector and SOCS3-SH2 mutant were 16.1 ± 3.8, 15.7 ± 3.5, respectively (p > 0.05, data not shown).

**Figure 5:**
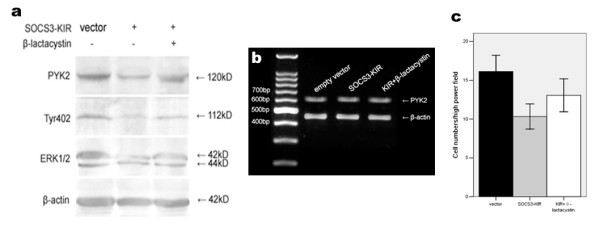
**Effects of SOCS3-KIR mutant on PYK2 expression, Tyr402 and ERK1/2 activations and migration of A549 cells**. (a) Down-regulations of PYK2 expression, Tyr402 and ERK1/2 phosphorylations by SOCS3-KIR mutant. A549 cells were transfected with either vector or the SOCS3-KIR mutant for 48 h, and cell lysates were subjected to western blot using the indicated antibodies. Cells pretreated with β-lactacystin showed that PYK2 expression, Tyr402 and ERK1/2 phosphorylations were restored to some extent. (b) PYK2 mRNA levels were unaffected regardless of transfection or not. The amplified PYK2 products were 629 bp in length. β-actin amplification demonstrated the consistency of PT-PCR. (c) Cell migration inhibited by the SOCS3-KIR mutant was statistically significant, and β-lactacystin pretreatment restored cell migration to some degree. The values are means of three replicates.

Cells with the KIR domain inactivated SOCS3 showed reduced PYK2 expression, as well as PYK2 and ERK1/2 phosphorylations (p < 0.05, Fig. 5a), which were in parallel with inhibited cell migration. The numbers of migrated cells with vector or SOCS3-KIR mutant transfection, and β-lactacystin pretreatment were 16.1 ± 3.8, 10.3 ± 2.9 and 13.8 ± 3.6, respectively (p < 0.05, Fig. 5c). To further determine whether the suppressed cell migration was induced by SOCS-box-mediated proteasome-dependent degradation of PYK2, pretreatment with β-lactacystin was performed in transfected cells, and PYK2 mRNA levels were also examined. We found that β-lactacystin elevated PYK2 expression, and the number of migrated cells also increased. However, PYK2 mRNA levels were unchanged (p > 0.05, Fig. 5b). These results indicated that the inhibited migration of A549 cells was possibly correlated with proteasome-mediated degradation of PYK2.

**Figure 6:**
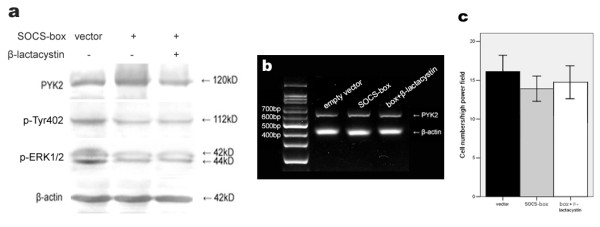
**Effects of SOCS-box mutant on PYK2 expression, Tyr402 and ERK1/2 activations and cell migration**. (a) Decreased PYK2 and ERK1/2 phosphorylations by SOCS-box mutant with the same PYK2 expression. A549 cells were vector- or the SOCS-box mutant-transfected for 48 h, and cell lysates were analysed by western blot using the indicated antibodies. (b) PYK2 mRNA levels were unaffected regardless of transfection or not. The amplified PYK2 products were 629 bp in length. β-actin amplification demonstrated the consistency of PT-PCR. (c) Cell migration suppressed by the SOCS-box mutant was not statistically significant. The values are means of three replicates.

Next, A549 cells were transfected with SOCS-box mutant. Compared with the vector alone-transfected cells, Tyr402 and ERK1/2 phosphorylations were inhibited (p < 0.05, Fig. 6a), despite that these cells expressed similar level of PYK2 as control cells (p > 0.05, Fig. 6a). Pretreatment with β-lactacystin was also performed in transfected cells and no effects were detected on PYK2 expression, Tyr402 and ERK1/2 activations (p > 0.05, Fig. 6a). PYK2 mRNA levels were unchanged as well (p > 0.05, Fig. 6b). The numbers of migrated cells with vector or SOCS-box mutant transfection, and β-lactacystin pretreatment were 16.1 ± 3.8, 13.8 ± 4.5 and 14.2 ± 4.2, respectively (p > 0.05, Fig. 6c). The SOCS-box inactivated mutant failed to decrease PYK2 expression and inhibit cell migration. The suppressed phosphorylations of Tyr402 and ERK1/2 were possibly due to the kinase inhibitory activity of SOCS3.

### Interactions of exogenous SOCS3 mutants with PYK2 in A549 cells

**Figure 7:**
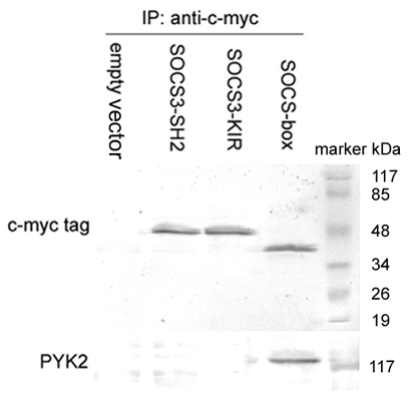
**Interactions of exogenously expressed SOCS3 mutants with PYK2 in A549 cells**. A549 cells were transfected with either vector or SOCS3 mutants with distinct functional domains deleted, and pretreated with β-lactacystin 4 h before transfection until the cell lysates were subjected to anti-myc tag immunoprecipitations and analysed by western blot using the anti-PYK2 antibody. The co-immunoprecipitation product was clearly observed in the SOCS-box mutant transfected cells. The results are representative of three individual experiments.

We further investigated whether exogenously expressed SOCS3 mutants interacted with PYK2 in the transfected A549 cells. As shown in Fig. 7, exogenous SOCS3-SH2, -KIR and -box mutants were detected in A549 cells, as indicated by the myc tag. PYK2 was co-immunoprecipitated with exogenous myc tag, which is clearly seen in SOCS-box mutant-transfected A549 cells but not in vector control cells. However, neither SOCS3-SH2 nor -KIR mutant/PYK2 co-IP product was observed in transfected A549 cells (Fig. 7). These results collectively suggested that the SH2 and KIR domains were essential for SOCS3 interacting with PYK2, and the formation of SOCS3/PYK2 complex may result in inhibited migration of A549 cells.

## Discussion

Although much progress has been made on elucidating the PYK2 downstream signaling pathways, relatively little is known about the regulatory mechanisms of PYK2. Our studies in this report demonstrated that SOCS3 might have a previously unidentified role in negatively regulating PYK2 function. The expression of SOCS3 is decreased due to aberrant methylation, and its transcriptional silencing is associated with malignant tumor behaviors [27]. Human SOCS3 gene is composed of the noncoding exon 1, intron 1 and exon 2. In our studies, the methylation- and non-methylation-specific primers were used to detect methylation status of SOCS3 in the exon 1, intron 1 and exon 2 in A549 cells respectively. We found SOCS3 methylation in the exon 2, which lies in a region of 92–250 bp downstream to the translation start site, although no detectable methylation was found in the promotor region containing the noncoding exon 1 and intron 1, which was consistent with the previous findings [28]. This demonstrated that methylation in the exon 2 was associated with the decrease of SOCS3 expression in A549 cells. The demethylation agent 5-aza-2'-deoxycytidine reactivated SOCS3, which further indicated that SOCS3 expression was attenuated by the associated methylation. Taken together, the exon 2 of SOCS3 appeared to be a crucial target for methylation in A549 cells. However, the significance of methylation in exons has not been elucidated.

It has been shown that SOCS3 transfection inhibited proliferation of A549 cells [15]. We therefore examined the effect of SOCS3 up-regulation on migration of A549 cells. In our studies, we found that SOCS3 restoration inhibited cell migration. The negative regulation of SOCS3 was previously reported in PDGF-induced fibroblast [29] and HGF-promoted keratinocyte migration [30], which was mediated by STAT3. However, STAT3 pathway may not be involved in SOCS3 methylation-induced migration of A549 cells, because STAT3 binding sites [25] are not included in the exon 2 of SOCS3. These data supported that SOCS3 methylation-associated inactivation might facilitate cell motility, and SOCS3 reactivation by 5-aza-2'-deoxycytidine treatment inhibited migration of A549 cells.

A growing number of reports have demonstrated that PYK2 plays a significant role in development of various tumors. In metastatic glioma cells and hepatocellular carcinoma, PYK2 expression is significantly elevated [31,32]. PYK2 becomes Tyr402-phosphorylated in invasive breast cancer [33] and SCLC cells [34], and PYK2-associated ERK1/2 activation participates in up-regulating the adhesive ability of PCa cells [35]. Pyk2 becomes activated by a number of cytokines that are also known to regulate SOCS3, such as SDF-1/CXCL12 [2,18]. As a negative regulator for PYK2 in T cells, SOCS3 showed migration suppression activity [36]. We also notice that SOCS3 binds to the phosphorylated Tyr397 of FAK, a close homolog of PYK2, and inhibits its kinase activity or induces degradation by proteasome pathway [19]. Thus, similar mechanisms of interaction and inhibition could apply to PYK2. We found that PYK2 expression, Tyr402 and ERK1/2 phosphorylations were inhibited with the increasing expression of SOCS3 by the treatment of 5-aza-2'-deoxycytidine. PYK2 seemed to be down-regulated by proteasome-mediated degradation, because pretreatment with β-lactacystin restored PYK2 expression, Tyr402 and ERK1/2 phosphorylations to some extent and PYK2 mRNA levels were unchanged no matter whether treatment or not. As we concluded, inhibited migration of A549 cells was associated with the reduced PYK2 expression, Tyr402 and ERK1/2 phosphorylations after SOCS3 restoration.

We further examined the effects of SOCS3 mutants on cell motility, and the associated PYK2 expression, Tyr402 and ERK1/2 phosphorylations. When SOCS3-SH2 mutant exogenously expressed in A549 cells, the PYK2 expression, Tyr402 and ERK1/2 phosphorylations, as well as cell migration were unchanged regardless of whether transfection or not. Moreover, the interaction of exogenous SOCS3-SH2 mutant with PYK2 was not detected. These findings indicated that the SH2 region inactivated mutant deprived SOCS3 of the ability to down-regulate PYK2-associated signaling and function in A549 cells. Therefore, the SH2 domain is crucial for SOCS3 regulating PYK2.

Compared with control cells, A549 cells with SOCS3-KIR mutant showed decreased PYK2 expression. Tyr402 and ERK1/2 phosphorylations, as well as cell mobility. Pretreatment with β-lactacystin restored PYK2 expression, Tyr402 and ERK1/2 phosphorylations, and cell migration to some extent. We proposed that the binding of SOCS3 with PYK2 via SH2 domain and SOCS-box-mediated proteasome-dependent degradation of PYK2 led to the suppressed migration of transfected A549 cells. The roles of SOCS3-KIR mutant were observed definitely, however the potential interaction as we presumed between the exogenous SOCS3-KIR mutant and PYK2 was not detected. We supposed that SOCS3-KIR mutant interacted with PYK2 principally by the central SH2 domain, generating the inhibition effects, however the binding was a functional interaction, unstable and readily reversible, and the effects would be transient without the help of KIR domain, thus could not be detected by immunoprecipitation in vitro. Therefore we concluded that the KIR domain is possibly required for a more stable binding of SOCS3 with PYK2 based on the SH2 domain. Further investigation is required to demonstrate our point of view.

The SOCS-box mutant had no influence on PYK2 expression, but Tyr402 and ERK1/2 phosphorylations were reduced in contrast to control cells. The migration of transfected A549 cells was also inhibited. These data denoted that the SOCS-box inactivated mutant down-regulated PYK2-associated signaling and function possibly by binding to PYK2 via the SH2 domain and inhibiting PYK2 activation by the KIR domain. PYK2 was detected in cell lysates immunoprecipitated with anti-myc tag antibody, which suggested a direct interaction between SOCS-box mutant and PYK2. Taken together, the stable interaction between exogenous SOCS3 and PYK2 is dependent on the SH2 and KIR domains of SOCS3, which results in the observed decrease in PYK2 expression, leading to the subsequent Tyr402 and ERK1/2 inactivations.

Studies have demonstrated that Tyr402 phosphorylation of PYK2 leads to binding of the SH2 domain and activation of Src [37], or integration of the SH2 domain of CHK and inhibition of breast cancer cell migration [38]. Our results are suggestive of an important role of SOCS3 in blocking Tyr402 phosphorylation. However, it remains to be determined whether Tyr402 mediates the interaction between SOCS3 and PYK2. Moreover, the precise role of SOCS3 in regulating migration-associated cytoskeleton proteins downstream of PYK2 has not been characterized in relation to lung cancer metastasis. Taken together, our results indicate that SOCS3 interacts with PYK2 and inhibits PYK2-associated migration of A549 cells. Further research is needed to clarify the pathological significance of PYK2 expression and interaction with SOCS3 in various signaling pathways. Studies of SOCS3 may foster new anti-chemotactic approaches to suppress lung cancer metastasis.

## Conclusion

Our data suggested that SOCS3 negatively regulates cell motility and decreased SOCS3 induced by methylation may confer a migration advantage to A549 cells. Exogenous SOCS3 interacted with PYK2, and both the SH2 and KIR domains of SOCS3 contributed to PYK2 binding. These results also suggest a negative role of SOCS3 in PYK2 signaling, and a previously unidentified regulatory mechanism for PYK2 function.

## Competing interests

The authors declare that they have no competing interests.

## Authors' contributions

SZ initiated the research, carried out the experiments and wrote the manuscript, DG contributed to the paper translation, LJ and QZ gave experimental instructions, XQ helped with the experimental design and gave funding support, and EW gave critical review of the manuscript. All authors read and approved the final manuscript.

## Pre-publication history

The pre-publication history for this paper can be accessed here:



## Supplementary Material

Additional file 1 Statistical analysis of O.D. differences between groups with various treatments. O.D. values of specific bands were measured using the Image J software. Statistical analysis was used to evaluate the differences of O.D. values between groups with various treatments. The results are representative of three individual experiments and data are expressed as mean ± SD. Click here for file

## References

[B1] Shi CS, Kehrl JH (2004). Pyk2 amplifies epidermal growth factor and c-Src-induced Stat3 activation. J Biol Chem.

[B2] Massa A, Casagrande S, Bajetto A, Porcile C, Barbieri F, Thellung S, Arena S, Pattarozzi A, Gatti M, Corsaro A, Robello M, Schettini G, Florio T (2006). SDF-1 controls pituitary cell proliferation through the activation of ERK1/2 and the Ca2+-dependent, cytosolic tyrosine kinase Pyk2. Ann N Y Acad Sci.

[B3] Horst EH van der, Weber I, Ullrich A (2005). Tyrosine phosphorylation of PYK2 mediates heregulin-induced glioma invasion: novel heregulin/HER3-stimulated signaling pathway in glioma. Int J Cancer.

[B4] Ridyard MS, Sanders EJ (2000). Comparison between the in vivo and vitro expression of three adhesion-signaling proteins in embryonic cells. Cell Biol Int.

[B5] Okigaki M, Davis C, Falasca M, Harroch S, Felsenfeld DP, Sheetz MP, Schlessinger J (2003). Pyk2 regulate multiple signaling events crucial for macrophage morphology and migration. Proc Natl Acad Sci USA.

[B6] de Amicis F, Lanzino M, Kisslinger A, Calì G, Chieffi P, Andò S, Mancini FP, Tramontano D (2006). Loss of proline-rich tyrosine kinase 2 function induces spreading and motility of epithelial prostate cells. J Cell Physiol.

[B7] Wei L, Yang Y, Zhang X, Yu Q (2004). Altered regulation of Src upon cell detachment protects human lung adenocarcinoma cells from anoikis. Oncogene.

[B8] Lipinski CA, Tran NL, Bay C, Kloss J, McDonough WS, Beaudry C, Berens ME, Loftus JC (2003). Differential role of proline-rich tyrosine kinase 2 and focal adhesion kinase in determining glioblastoma migration and proliferation. Mol Cancer Res.

[B9] Owen KA, Pixley FJ, Thomas KS, Vicente-Manzanares M, Ray BJ, Horwitz AF, Parsons JT, Beggs HE, Stanley ER, Bouton AH (2007). Regulation of lamellipodial persistence, adhesion turnover, and motility in macrophages by focal adhesion kinase. J Cell Biol.

[B10] Allingham MJ, van Buul JD, Burridge K (2007). ICAM-1-mediated, Src- and Pyk2-dependent vascular endothelial cadherin tyrosine phosphorylation is required for leukocyte transendothelial migration. J Immunol.

[B11] Rucci N, Ricevuto E, Ficorella C, Longo M, Perez M, Di Giacinto C, Funari A, Teti A, Migliaccio S (2004). In vivo bone metastases, osteoclastogenic ability, and phenotypic characterization of human breast cancer cells. Bone.

[B12] Melendez J, Turner C, Avraham H, Steinberg SF, Schaefer E, Sussman MA (2004). Cardiomyocyte apoptosis triggered by RAFTK/pyk2 via Src kinase is antagonized by paxillin. J Biol Chem.

[B13] Schaeffer M, Schneiderbauer M, Weidler S, Tavares R, Warmuth M, de Vos G, Hallek M (2001). Signaling through a novel domain of gp130 mediates cell proliferation and activation of Hck and Erk kinases. Mol Cell Biol.

[B14] Zrihan-Licht S, Fu Y, Settleman J, Schinkmann K, Shaw L, Keydar I, Avraham S, Avraham H (2000). RAFTK/Pyk2 tyrosine kinase mediates the association of p190 RhoGAP with RasGAP and is involved in breast cancer cell invasion. Oncogene.

[B15] Yu ZB, Min JX, Bai L, Yao K, Zhang GQ (2004). Effects of transfected SOCS3 gene on proliferation of human lung adenocarcinoma cell line A549. Ai Zheng.

[B16] Bai L, Yu Z, Qian G, Qian P, Jiang J, Wang G, Bai C (2006). SOCS3 was induced by hypoxia and suppressed STAT3 phosphorylation in pulmonary arterial smooth muscle cells. Respir Physiol Neurobiol.

[B17] Sun R, Jaruga B, Kulkarni S, Sun H, Gao B (2005). IL-6 modulates hepatocyte proliferation via induction of HGF/p21cip1: regulation by SOCS3. Biochem Biophys Res Commun.

[B18] Le Y, Zhu BM, Harley B, Park SY, Kobayashi T, Manis JP, Luo HR, Yoshimura A, Hennighausen L, Silberstein LE (2007). SOCS3 proein developmentally regulates the chemokine receptor CXCR4-FAK signaling pathway during B lymphopoiesis. Immunity.

[B19] Liu E, Côté JF, Vuori K (2003). Negative regulation of FAK signaling by SOCS proteins. EMBO J.

[B20] Tokita T, Maesawa C, Kimura T, Kotani K, Takahashi K, Akasaka T, Masuda T (2007). Methylation status of the SOCS3 gene in human malignant melanomas. Int J Oncol.

[B21] Tischoff I, Hengge UR, Vieth M, Ell C, Stolte M, Weber A, Schmidt WE, Tannapfel A (2007). Methylation of SOCS-3 and SOCS-1 in the carcinogenesis of Barrett's adenocarcinoma. Gut.

[B22] Sasaki A, Yasukawa H, Suzuki A, Kamizono S, Syoda T, Kinjyo I, Sasaki M, Johnston JA, Yoshimura A (1999). Cytokine-inducible SH2 protein-3 (CIS3/SOCS3) inhibits Janus tyrosine kinase by binding through the N-terminal kinase inhibitory region as well as SH2 domain. Genes Cells.

[B23] Bergamin E, Wu J, Hubbard SR (2006). Structural basis for phosphotyrosine recognition by suppressor of cytokine signaling-3. Structure.

[B24] Shouda T, Hiraoka K, Komiya S, Hamada T, Zenmyo M, Iwasaki H, Isayama T, Fukushima N, Nagata K, Yoshimura A (2006). Suppression of IL-6 production and proliferation by blocking STAT3 activation in malignant soft tissue tumor cells. Cancer Lett.

[B25] Niwa Y, Kanda H, Shikauchi Y, Saiura A, Matsubara K, Kitagawa T, Yamamoto J, Kubo T, Yoshikawa H (2005). Methylation silencing of SOCS-3 promotes cell growth and migration by enhancing JAK/STAT and FAK signalings in human hepatocellular carcinoma. Oncogene.

[B26] Herman JG, Graff JR, Myöhänen S, Nelkin BD, Baylin SB (1996). Methylation-specific PCR: a novel PCR assay for methylation status of CpG islands. Proc Natl Acad Sci USA.

[B27] Isomoto H, Mott JL, Kobayashi S, Werneburg NW, Bronk SF, Haan S, Gores GJ (2007). Sustained IL-6/STAT-3 signaling in cholangiocarcinoma cells due to SOCS-3 epigenetic silencing. Gastroenterology.

[B28] He B, You L, Uematsu K, Zang K, Xu Z, Lee AY, Costello JF, McCormick F, Jablons DM (2003). SOCS-3 is frequently silenced by hypermethylation and suppresses cell growth in human lung cancer. Proc Natl Acad Sci U S A.

[B29] Nagai H, Tokumaru S, Sayama K, Shirakata Y, Hanakawa Y, Hirakawa S, Dai X, Tohyama M, Yang L, Hashimoto K (2007). Suppressor of cytokine signaling 3 negative regulation of signal transducer and activator of transcription 3 in platelet-derived growth factor-induced fibroblast migration. J Dermatol.

[B30] Tokumaru S, Sayama K, Yamasaki K, Shirakata Y, Hanakawa Y, Yahata Y, Dai X, Tohyama M, Yang L, Yoshimura A, Hashimoto K (2005). SOCS3/CIS3 negative regulation of STAT3 in HGF-induced keratinocyte migration. Biochem Biophys Res Commun.

[B31] Lipinski CA, Tran NL, Menashi E, Rohl C, Kloss J, Bay RC, Berens ME, Loftus JC (2005). The tyrosine kinase pyk2 promotes migration and invasion of glioma cells. Neoplasia.

[B32] Sun CK, Ng KT, Sun BS, Ho JW, Lee TK, Ng I, Poon RT, Lo CM, Liu CL, Man K, Fan ST (2007). The significance of proline-rich tyrosine kinase 2 (PYK2) on hepatocellular carcinoma progression and recurrence. Br J Cancer.

[B33] Fernandis AZ, Prasad A, Band H, Klösel R, Ganju RK (2004). Regulation of CXCR4-mediated chemotaxis and chemoinvasion of breast cancer cells. Oncogene.

[B34] Maulik G, Kijima T, Ma PC, Ghosh SK, Lin J, Shapiro GI, Schaefer E, Tibaldi E, Johnson BE, Salgia R (2002). Modulation of the c-Met/Hepatocyte growth factor pathway in small cell lung cancer. Clin Cancer Res.

[B35] Yuan TC, Lin FF, Veeramani S, Chen SJ, Earp HS, Lin MF (2007). ErbB-2 via PYK2 upregulates the adhesive ability of androgen receptor-positive human prostate cancer cells. Oncogene.

[B36] Zanin-Zhorov A, Tal G, Shivtiel S, Cohen M, Lapidot T, Nussbaum G, Margalit R, Cohen IR, Lider O (2005). Heat shock protein 60 activates cytokine-associated negative regulator suppressor of cytokine singaling 3 in T cells: effects on signaling, chemotaxis, and inflammation. J Immunol.

[B37] Dikic I, Tokiwa G, Lev S, Courtneidge SA, Schlessinger J (1996). A role for Pyk2 and Src in linking G-protein-coupled receptors with MAP kinase activation. Nature.

[B38] McShan GD, Zagozdzon R, Park SY, Zrihan-Licht S, Fu Y, Avraham S, Avraham H (2002). Csk homologous kinase associates with RAFTK/Pyk2 in breast cancer cells and negatively regulates its activation and breast cancer cell migration. Int J Oncol.

