# Supplementary material for: SOCS3 inhibiting migration of A549 cells correlates with PYK2 signaling in vitro
Source: BMC Cancer. 2008 May 28;8:150. doi: 10.1186/1471-2407-8-150 (PMC2429913; doi:10.1186/1471-2407-8-150)
Supplement: Additional file 1 — Statistical analysis of O.D. differences between groups with various treatments. O.D. values of specific bands were measured using the Image J software. Statistical analysis was used to evaluate the differences of O.D. values between groups with various treatments. The results are representative of three individual experiments and data are expressed as mean ± SD. [file 1471-2407-8-150-S1.doc]

| Items | Groups | O.D. values (Mean±SD) | P value |
| --- | --- | --- | --- |
| PYK2 expression | HBE  A549 | 56.03±1.87  72.50±1.06 | p<0.05 |
|  |  |  |  |
| p-Tyr402 | HBE  A549 | 23.49±2.84  64.60±1.63 | p<0.05 |
|  |  |  |  |
| p-ERK1/2 | HBE  A549 | 62.94±2.36  117.89±1.08 | p<0.05 |
|  |  |  |  |
| SOCS3 expression | HBE  A549 | 76.02±2.35  56.54±1.58 | p<0.05 |
|  | untreated A549  5-aza treated A549 | 51.58±1.10  67.58±0.55 | p<0.05 |
|  |  |  |  |
| PYK2 expression | untreated  5-aza treated  β-lactacystin treated | 70.78±2.05  41.67±1.67  51.68±1.14 | p<0.05 |
|  |  |  |  |
|  | empty vector  SOCS3-SH2 transfection  β-lactacystin treated | 74.04±3.69  74.70±2.61  72.47±2.43 | p>0.05 |
|  |  |  |  |
|  | empty vector  SOCS3-KIR transfection  β-lactacystin treated | 79.12±1.44  58.94±1.37  75.49±1.46 | P<0.05 |
|  |  |  |  |
|  | empty vector  SOCS-box transfection  β-lactacystin treated | 74.04±3.69  74.53±3.53  72.41±2.56 | p>0.05 |
|  |  |  |  |
| p-Tyr402 | untreated  5-aza treated  β-lactacystin treated | 61.72±2.07  37.23±2.22  43.66±1.41 | p<0.05 |
|  |  |  |  |
|  | empty vector  SOCS3-SH2 transfection  β-lactacystin treated | 69.83±0.70  70.79±1.39  70.21±1.63 | p>0.05 |
|  |  |  |  |
|  | empty vector  SOCS3-KIR transfection  β-lactacystin treated | 67.20±0.24  39.30±2.65  49.21±0.81 | P<0.05 |
|  |  |  |  |
|  | empty vector  SOCS-box transfection  β-lactacystin treated | 69.83±0.70  45.08±1.69  44.62±2.14 | p<0.05 |
|  |  |  |  |
| p-ERK1/2 | untreated  5-aza treated  β-lactacystin treated | 123.02±2.96  94.93±0.99  106.01±1.66 | P<0.05 |
|  |  |  |  |
|  | empty vector  SOCS3-SH2 transfection  β-lactacystin treated | 125.31±2.61  120.25±4.27  123.70±2.14 | p>0.05 |
|  |  |  |  |
|  | empty vector  SOCS3-KIR transfection  β-lactacystin treated | 126.30±4.19  72.49±2.74  89.75±1.41 | p<0.05 |
|  |  |  |  |
|  | empty vector  SOCS-box transfection  β-lactacystin treated | 135.34±2.67  86.51±3.59  84.74±0.65 | p<0.05 |
|  |  |  |  |
| PYK2 mRNA expression | untreated  5-aza treated  β-lactacystin treated | 80.91±1.39  81.01±0.99  81.30±2.11 | p>0.05 |
|  |  |  |  |
|  | empty vector  SOCS3-SH2 transfection  β-lactacystin treated | 97.22±3.30  98.66±2.59  96.78±3.74 | p>0.05 |
|  |  |  |  |
|  | empty vector  SOCS3-KIR transfection  β-lactacystin treated | 116.32±2.87  117.21±4.40  115.30±4.78 | p>0.05 |
|  |  |  |  |
|  | empty vector  SOCS-box transfection  β-lactacystin treated | 117.29±3.44  116.21±4.46  117.08±3.44 | p>0.05 |
